# Influence of Calcium Binding on Conformations and Motions of Anionic Polyamino Acids. Effect of Side Chain Length

**DOI:** 10.3390/polym12061279

**Published:** 2020-06-03

**Authors:** Dmitry Tolmachev, Natalia Lukasheva, George Mamistvalov, Mikko Karttunen

**Affiliations:** 1 Institute of Macromolecular Compounds, Russian Academy of Sciences, Bolshoy pr. 31, 199004 St. Petersburg, Russia; luk@imc.macro.ru; 2 Faculty of Physics, St. Petersburg State University, Petrodvorets, 198504 St. Petersburg, Russia; mamistvalov.georgii@gmail.com; 3 Department of Chemistry, the University of Western Ontario, 1151 Richmond Street, London, ON N6A 5B7, Canada; 4 Department of Applied Mathematics, the University of Western Ontario, 1151 Richmond Street, London, ON N6A 5B7, Canada; 5 The Centre of Advanced Materials and Biomaterials Research, the University of Western Ontario, 1151 Richmond Street, London, ON N6A 5B7, Canada

**Keywords:** mineralization, salt solutions, poly(amino acids), poly-(α-l glutamic acid), poly-(α-l aspartic acid), molecular dynamic simulation, Hamiltonian replica exchange

## Abstract

Investigation of the effect of CaCl_2_ salt on conformations of two anionic poly(amino acids) with different side chain lengths, poly-(α-l glutamic acid) (PGA) and poly-(α-l aspartic acid) (PASA), was performed by atomistic molecular dynamics (MD) simulations. The simulations were performed using both unbiased MD and the Hamiltonian replica exchange (HRE) method. The results show that at low CaCl_2_ concentration adsorption of Ca^2+^ ions lead to a significant chain size reduction for both PGA and PASA. With the increase in concentration, the chains sizes partially recover due to electrostatic repulsion between the adsorbed Ca^2+^ ions. Here, the side chain length becomes important. Due to the longer side chain and its ability to distance the charged groups with adsorbed ions from both each other and the backbone, PGA remains longer in the collapsed state as the CaCl_2_ concentration is increased. The analysis of the distribution of the mineral ions suggests that both poly(amino acids) should induce the formation of mineral with the same structure of the crystal cell.

## 1. Introduction

Polyelectrolytes, such as anionic poly(amino acids), are widely used in diverse applications including water treatment and purification [1], anticorrosion agents [2], drug delivery [3,4,5,6] and tissue engineering [7,8,9,10,11]. Biodegradable and biocompatible anionic poly(amino acids) are attractive due to their comparative cheapness and ease of high-volume manufacturing. From the practical point of view, one of the key features of polyelectrolytes is their ability to chelate cations. For example, poly-(α-l aspartic acid) (PASA) and poly-(α-l glutamic acid) (PGA) are commonly used to guide nucleation and crystal growth in calcium containing minerals [8,12,13]. PASA and PGA have successfully been used in the regulation of mineralization in organic matrices in the synthesis of scaffolds for bone tissue [8,9,10]; anionic poly(amino acids) (both PASA and PGA) and short peptides can be grafted onto surfaces to increase mineralization [14,15,16]. On the other hand, in some cases, anionic poly(amino acids) are used for the opposite purpose, that is, to inhibit calcification [17,18,19]. PASA is commonly used in the synthesis of organomineral composite materials as polymer agent for the delivery of minerals to organic matrices [8,13]. It is typically assumed that PASA forms a polymer-induced liquid-like precursor, which is able to penetrate into the pores of the organic matrix. This results in higher quality final structures.

**Figure 1:**
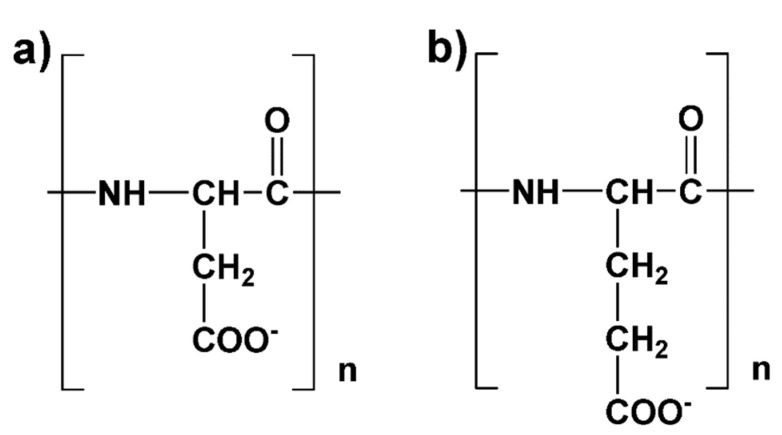
The chemical structures of (**a**) poly-(α-l aspartic acid) (PASA) and **(b**) poly-(α-l glutamic acid) (PGA) molecules. The degree of polymerization (n) in the simulations was 32.

Conformations of polyelectrolyte chains play an important functional role [20,21,22]. Adsorption of multivalent cations on a polyelectrolyte chain may lead to significant changes in their sizes and conformations [23,24,25,26,27,28]. In particular, Thula et al. have shown the critical role of polyelectrolyte chemical structure [8]. Their work demonstrates a significant difference in mineralization of organic matrices when different polyelectrolyte agents are used. They observed differences even for systems with PGA and PASA, which differ only by their side chain length (difference of one methylene group, Figure 1). Similarly, Picker et al. have demonstrated that aspartic and glutamic acids have qualitatively different effects on the process of calcium carbonate crystallization [12] and Grohe et al. have shown that the effect of PGA on calcium oxalate monohydrate growth depends strongly on PGA concentration [29].

The influence of salt on a polyelectrolyte chain can be described through the screening of electrostatic interactions [30,31], the formation of ion bridges between charged groups [28,32,33], and in some cases even charge inversion can occur [34,35]. In the case of the α-forms of PGA and PASA, the carboxyl groups—the terminal groups of the side chains—carry charges (Figure 1). The influence of ion bridges on backbone conformation generally depends on the length of the side chain [36,37,38]. Multivalent ions can cause changes in the sizes of the polyelectrolyte chains and alter their local conformations. It has been shown that Ca^2+^ ions can induce specific secondary structures in different peptides not only by screening electrostatics but also by forming ion bridges between anionic monomers [39,40,41].

We have performed atomistic MD simulations to investigate the effects of CaCl_2_ salt on conformations of PASA and PGA. However, simulations of systems with explicit charges are not trivial and there are several issues, both practical and fundamental, related to strong electrostatic interactions [42,43,44,45]. One of the main practical issues is the overbinding of charged atoms in classical MD force fields due to the absence of electronic polarizability [46,47,48,49]. One possible remedy for this problem is to include polarizability directly in the force field [50,51,52]. However, polarizable force fields are difficult to parameterize, are not available for all compounds and their use involves a considerably larger computational cost [53,54,55]. Another solution to this problem is to add corrections into the electrostatic interactions of the existing classical force fields [47,48,49,56]. Each of the corrections has its own advantages and disadvantages, and the choice between them should be determined by the details of the particular system [49,57].

In addition to the above, there is the fundamental and practical matter that interactions between explicit charges can be very strong especially in multivalent systems. Strong interactions cause slowing down of the motions and rearrangements in the system. For example, lifetimes of ion bridges in proteins [58] and between charged groups in polyelectrolytes [59] could reach 1 s, which is about six orders of magnitude longer than current typical MD simulations and three orders of magnitude longer than the current longest MD simulation performed on a special-purpose computer [60]. This leads to the problem of correct sampling in classical MD [45].

Several advanced methods based on overcoming high-energy barriers such as replica exchange [61], metadynamics [62,63,64] and its many variants have been developed [65,66,67,68]. The features of these methods are well described in several recent reviews [68,69,70,71]. In some cases, it may be difficult to choose the best collective variables [67,70]; there is the risk that the chosen set of collective variables does not provide the true spectrum of metastable subensembles due to the presence of hidden energy barriers. On the other hand, parallel-tempering methods such as replica exchange are able to broadly sample the conformational space. One drawback is the high computational cost. In systems with a large number of degrees of freedom, a large number of replicas is required [68]. Moreover, in the cases when energy barriers have entropic nature, a simple increase in temperature may not necessarily improve sampling. To solve these problems a modification called Hamiltonian replica exchange (HRE) was developed [72]. HRE methods are based on varying broad types of simulation parameters including force field parameters. It helps to overcome energy barriers (including ones with entropic nature) using a low number of replicas.

Some of the above advanced sampling methods have been successfully used for simulations of polyelectrolytes in CaCl_2_ solutions. For example, metadynamics has been used to investigate the interactions of calcium with glutamic residues [56] and polyacrylates [73]. The replica exchange technique has been used to study the structural properties of glutamic acid oligomers in CaCl_2_ solutions [74]. Recently, Lemke et al. suggested a state-of-the-art approach for simulations of aspartic acid trimers in CaCl_2_ solutions based on the HRE method [75]. We have adopted this approach for the current research. In addition to studying polymer conformations, we provide a detailed description of the performance of the HRE method and give practical recommendations for simulations of similar systems.

## 2. Model Description

MD simulations were performed using short chain poly(amino acids): poly-α-l-(glutamic acid) (PGA) and poly-α-l-(aspartic acid) (PASA), see Figure 1 for their structures. The degree of polymerization was 32 and all carboxyl groups were deprotonated. The simulation box included only one poly(amino acid) molecule surrounded by CaCl_2_ solution. The simulation box sizes were chosen to ensure the absence of possible self-interaction through periodic boundary conditions. Box dimensions were approximately 7 nm × 7 nm × 7 nm. The effective concentration of the poly(amino acid) was approximately 0.005 mol/kg.

To maintain charge neutrality, typically Na^+^ or K^+^ ions were added as counterions. We have previously shown that the behavior of both PGA and PASA depends on the counterion type [57]. In particular, unless corrections are used, many force fields have an artificially strong attraction between Na^+^ ions and charged groups, which results in unphysical chain conformations [49,57,76,77,78]. This problem is relevant for different systems with explicit charges, including proteins [39], nucleic acids [76], and lipid membranes [77]. K^+^ ions are, however, better described in force fields, and therefore more suitable as counterions. To maintain charge neutrality, we used K^+^ ions. Six systems with different CaCl_2_ concentrations were studied for both PGA and PASA. The concentrations and the details regarding ions are shown in Table 1.

## 3. MD Simulation

### 3.1. MD Parameters

All simulations were performed with GROMACS 2018.1 [79] patched with PLUMED v.2.4.2 [80]. The isothermal-isobaric (NPT) ensemble at the temperature of 300 K and at 1 bar pressure was used utilizing the Nosé–Hoover thermostat [81] and the Parrinello–Rahman barostat [82]. The time step was set to 2 fs. Long-range electrostatic interactions were treated using the particle-mesh Ewald (PME) method [83] and bond lengths involving hydrogen atoms were constrained with the P-LINCS algorithm [84]. The Visual Molecular Dynamics (VMD) software [85] was used for visual trajectory and the creation of snapshots.

The CHARMM27 force field [86] was used for all simulations. We used the TIP3P [87] water model which is recommended for the CHARMM27 force field [86]. As already discussed above, it is well-known that electrostatic interactions in classical biological force fields are overvalued [39,46,47,49,51]. There are several approaches to address this problem. One of them, the so-called electronic continuum corrections (ECC), is a mean-field technique based on rescaling the charges to reproduce the shielding produced by electronic polarizability [48]. This approach has been shown to work well in simulations of biological systems [39,49]. However, in our previous paper we have shown that despite the fact that this approach has a firm physical basis, it could cause unpredictable conformational behavior of the chain [57]. Another approach called the non-bonded fix (NBFIX) is based on modifying the contact distances between the charged atoms to enforce a decrease in binding affinities. This approach has worked well in several cases [47,57], but these corrections may affect such properties as poly-coordination and lead to unrealistic behavior [88]. The NBFIX model for divalent ions includes a fixed solvation shell, which prevents adsorption of the ions on the polyelectrolyte chain [76,88], and hence it is not suitable for our purposes.

Another correction, especially for interactions between glutamic acids and Ca^2+^ ions, was suggested by Church et al. [56]. They varied the σ-parameter of the Lennard-Jones (LJ) potential,

(1) $$U\left(r\right)=4\varepsilon_{ij}^{AB}\left[{\left(\frac{\sigma_{ij}^{AB}}{r_{ij}^{AB}}\right)}^{12}-{\left(\frac{\sigma_{ij}^{AB}}{r_{ij}^{AB}}\right)}^{6}\right]$$

for the interaction between the Ca^2+^ ions and the oxygens of the carboxyl groups of the glutamic acid residue in simulations of peptides in CaCl_2_ solutions using the CHARMM22* force field [89]. In particular, they changed the σ-parameter from 0.2732 nm (unmodified CHARMM *σ*-parameter) to 0.281 nm. In Equation (1) $r_{ij}^{AB}$ is the distance between atoms *i* and *j* of types *A* and *B*, ε is the depth of the potential well, and *σ* is the distance at which the pair potential is zero. Their results showed that an increase from 0.273 to 0.281 nm in the σ-parameter leads to a correct representation of the interaction energy corresponding to experimental NMR data [90,91]. We used this correction for the interactions between the Ca^2+^ ions and the oxygens in our simulations. The final configurations of the 1 μs MD simulations of PGA and PASA in water were used as the initial structures for their simulations in CaCl_2_ solutions.

### 3.2. Results from Classical Unbiased MD Simulations

**Figure 2:**
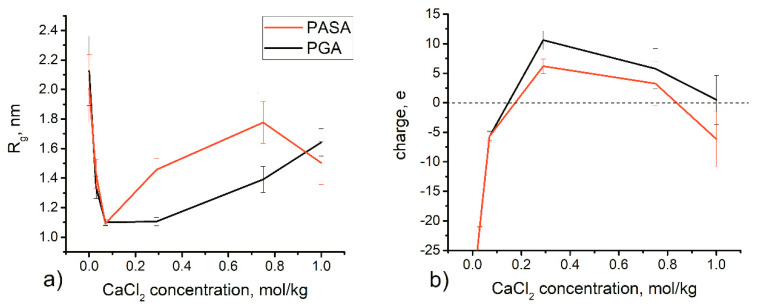
(**a**) Radius of gyration and (**b**) charge of the complex formed by the poly(amino acid) and adsorbed ions (both Ca^2+^ and Cl^−^) as a function of salt concentration. The horizontal dashed line corresponds to zero charge (exact charge neutralization).

We have reported results regarding simulations of PGA and PASA in water already earlier [57]. The addition of divalent CaCl_2_ salt in solution causes changes in chain conformations due to ion adsorption (Figure 2). During the first 200 ns, the number of ions adsorbed on the chain reached saturation (see Figure S1). Data analysis was performed after 200 ns of equilibration with CaCl_2_.

The radius of gyration and the effective charge of the complex formed by PGA and PASA and the adsorbed ions (both Ca^2+^ and Cl^−^) are shown as a function of CaCl_2_ concentration in Figure 2. The figure shows that R_g_ and charge of the complex depend non-linearly on CaCl_2_ concentration. The R_g_ curves have the typical shape that has been found for both flexible [25] and semiflexible [23] polyelectrolyte chains in multivalent salt solutions. This behavior is also in agreement with the theory of Kundagrami and Muthukumar [28].

In water, the PASA and PGA chains are fully extended due to the electrostatic repulsion between the carboxyl groups. When divalent Ca^2+^ ions are present in solution, they adsorb onto the carboxyl groups (so-called Manning or overcharging condensation [35,92]), and the electrostatic repulsion between these groups becomes reduced leading to a significant reduction in R_g_. With further increase in CaCl_2_ concentration, electrostatic screening becomes more important and eventually overcharging occurs. This leads to repulsion between the carboxyl groups with their adsorbed Ca^2+^ ions. As a result, R_g_ partially recovers. With an even further increase in CaCl_2_ concentration, the adsorbed Ca^2+^ ions form pairs with Cl^−^ ions leading to a decrease in the effective charge.

The overcharge of PGA is higher in comparison with PASA due to its longer side chains and ability to distance the charged groups with adsorbed ions from both each other and the backbone, which helps to compensate the electrostatic repulsion between them.

**Figure 3:**
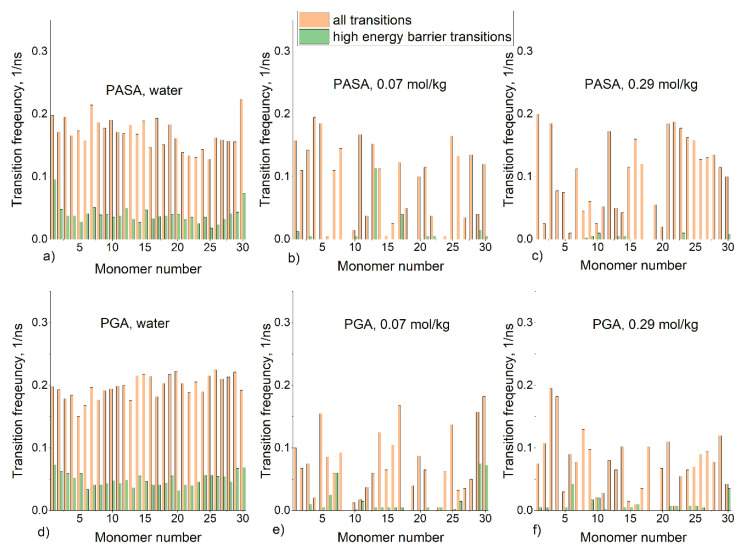
Transition frequencies of the φ and ψ dihedral angles for each monomer in the classical unbiased molecular dynamics (MD) simulations. Pink—all transitions, green—transitions through high-energy barriers. (**a**) PASA in water (**b**) PASA in 0.07 mol/kg CaCl_2_ solution, (**c**) PASA in 0.29 mol/kg CaCl_2_ solution, (**d**) PGA in water (**e**) PGA in 0.07 mol/kg CaCl_2_ solution, (**f**) PGA in 0.29 mol/kg CaCl_2_ solution.

The minimum value of R_g_ is observed at 0.07 mol/kg of CaCl_2_; in this case, the chains are almost fully neutralized. Further increase in concentration leads to an increase in R_g_ and a faster increase in R_g_ in the case of PASA. It can be proposed that the origin of this behavior lies in the different side-chain lengths of PASA and PGA (the only difference), and therefore the differences in the number of possible conformational states. However, due to the restrictions of conformational transitions caused by the long-lived Ca^2+^ bridges, the complete statistical ensemble cannot be reached by classical unbiased MD simulations, and thus, this result may be caused by poor statistical sampling. Figure 3 shows the frequency of the transitions of the φ and ψ dihedral angles for each monomer between the areas on the Ramachandran plot (Figure S2) related to PPII, 2.5_1_, left and right-handed α, and 3_10_ helices. Transitions which require overcoming the high-energy barrier (between areas of 3_10_+α helices and 2.5_1_ helix + PPII helices in Figure S2) are shown separately.

Figure 3a,d show that in water the frequencies of all transitions for all monomers are comparable. Transitions through high-energy barriers are at least one-third of the total number of transitions. With the addition of CaCl_2_, the motions of the backbone dihedral angles become very restricted and conformational transitions become nearly frozen. Most of the transitions through high-energy barriers are absent in almost all monomers. Transitions are allowed only for the monomers, which do not have bound Ca^2+^ ions.

To study the difference between the PASA and PGA chains in more detail, and to examine if the statistical ensembles obtained in the MD simulations are depleted, we employed the Hamiltonian replica exchange (HRE) technique [72]. This method is well-known as an effective way to simulate systems with high energy barriers [93,94].

## 4. Hamiltonian Replica Exchange Simulation

### 4.1. Hamiltonian Replica Exchange Parameters

Two concentrations were chosen for the HRE simulations, 0.07 mol/kg (as the concentration of the extreme value of R_g_) and 0.29 mol/kg (to investigate the different behaviors of the PGA and PASA chains observed in the unbiased MD simulations above). The final configurations of the unbiased MD simulations of PGA and PASA were used as the initial configurations in the HRE simulations.

The HRE is a modification of the replica exchange method [95]. It is based on parallel simulation of several replicas that differ by simulation conditions and have the ability to exchange states between each other. The classical replica exchange method is based on simulating the replicas at different temperatures. The exchanges between the replicas help the system to overcome the energy barriers and enrich the statistical ensemble.

However, Bulo et al. have shown that the classical replica exchange method is not effective for simulations of systems with ion bridges due to the entropic nature of their formation [96]. Instead, the HRE method is the preferred approach [72]. In HRE, the chosen force field parameters (instead of temperature) are varied to overcome energy barriers. One possibility, for example, is sequential changing of the Lennard-Jones parameters [93] or biasing of the dihedral potential for reduction of chain rigidity [97]. We adopted the HRE technique developed by Lemke, Peter and Kukharenko for simulations of aspartic acid trimers in CaCl_2_ solutions [75]. This technique is based on changing temperature, biasing the backbone dihedral angle potentials, and sequential changing (from replica to replica) of the Lennard-Jones parameters for the interactions between the Ca^2+^ ions and carboxyl groups. We call replicas which are closer to neutral replica (unbiased potential), the lowest replicas. Replicas with the most changed potentials are called the highest replicas. Next, we describe the method in more detail.

The optimized HRE parameters for biasing the backbone dihedral angle potentials were obtained by fitting the potential of the mean force obtained based on the dihedral distributions of PGA and PASA in pure water (Equation S1). The results of the fitting procedure are presented in Figure S3 and Table S1. The new potentials were implemented as tabulated potentials scaled by a factor α, which was varied in replicas from 0 to −1 in equal intervals (Table 2) and added to the regular dihedral potentials in the respective replicas as

(2) $$U\left(x\right)=\alpha \cdot \overset{15}{\underset{n=1}{\sum }}k_{n}\cdot \left(1+\cos\left(n*x-a_{n}\right)\right)$$

where α is the scaling factor, *x* is the dihedral angle, and $k_{n}$ and $a_{n}$ are parameters obtained from the fitting procedure (see Table S1). The resulting potentials for all replicas are shown in Figure S4.

**Figure 4:**
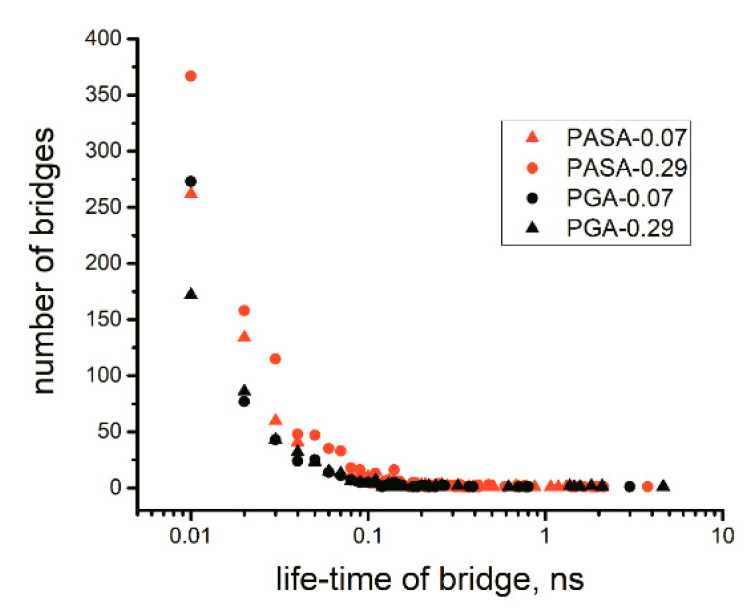
The distribution of calcium bridge lifetimes in the highest replica (replica number 21 in Table 2) without exchanges (data from the equilibration stage).

The addition of a bias potential is not enough to increase the sampling due to the presence of CaCl_2_ and the consequent formation of calcium bridges. To increase sampling, the Lennard-Jones parameters for the interactions between the Ca^2+^ ions and the carboxyl oxygens are also changed. The value of the σ-parameter (Equation (1)) was varied to ensure the absence of the long-lived calcium bridges with the highest replica [75]; following Bulo et al. [96] the value σ = 0.31 nm was chosen. The value of ε in Equation (1) was chosen to minimize the difference in the potential energy between the force fields of the neighboring replicas taking into account their different σ-parameters (see Table 2). The effectiveness of the chosen parameters is demonstrated in the next section (see Figure 4). Additionally, to accelerate the reorganization of solvent molecules and diffusion of ions, the temperature was increased from 300 (for the lowest replica) to 315 K (for the highest replica). Replica exchanges were attempted every 2 ps during the main simulation.

Lemke et al. showed that to obtain a complete statistical ensemble in their simulation of the trimer of aspartic acid in a CaCl_2_ solution, the length of the trajectory should be not less than 1 μs [75]. This is due to the formation of stable clusters consisting of ions and carboxyl groups and the consequent, slowing down of conformational changes. To avoid this problem, the parameters of the highest replica should ensure fast destruction of the contacts between the Ca^2+^ ions and the carboxyl groups. In addition, according to the definition of the replica exchange probability in Equation (3),

(3) $$P\left(x_{i}\to x_{j}\right)=\min\left[1,\;\;\exp\left(-\left(\frac{U_{i}\left(x_{j}\right)-U_{i}\left(x_{i}\right)}{k_{B}T_{i}}-\frac{U_{j}\left(x_{j}\right)-U_{j}\left(x_{i}\right)}{k_{B}T_{j}}\right)\right)\right]$$

a large difference in the parameters between neighboring replicas leads to a decrease in the frequency of exchanges between the replicas and, as a result, to a deceleration of the enrichment of the statistical ensemble. In Equation (3), *U(x_i_)* and *U(x_j_)* are the potential energies of the neighboring replicas *i* and *j*, and *U_i_* and *U_j_* are potential energies calculated for them. *T_i_* and *T_j_* are the corresponding temperatures. Thus, the number of replicas should be chosen according to the replica exchange probability (Equation (3)); in the simulations of trimers of aspartic acid, Lemke et al. used eight replicas [75]. In our case, we observed total absence of exchange between random replicas for all considered systems in our preliminary 100 ns simulation when using 11 replicas. This occurs because of the formation of large clusters due to high salt concentration and a relatively long chain. As a result, to accelerate the exchange between replicas, we used 21 replicas in the production run. The parameters are shown in Table 2. Each replica was equilibrated for 30 ns without exchanges between replicas. After equilibration, a 100 ns production was performed.

### 4.2. Analysis of Effectiveness of HRE Simulation

As discussed above, two processes should ensure the efficiency of HRE simulation: (1) The weakening of interactions between the Ca^2+^ ions and the carboxyl groups in higher replicas. This should be sufficient for the emergence of new states in the system and (2) successful exchanges of states between the replicas.

#### 4.2.1. Lifetime of Calcium Bridges

To verify the efficiency of the weakening of interactions between the Ca^2+^ ions and the carboxyl groups, the speed of calcium bridge destruction in the highest replica without exchanges (simulation on the equilibration stage) was analyzed in terms of their lifetimes; the details are described in Supporting Information (Figure S5) and the lifetimes are shown in Figure 4.

The initial configurations of the systems included a large number of calcium bridges because the final configurations of the systems obtained from the unbiased classical MD simulations contain them. In the classical MD simulations, the lifetimes were higher than the simulation times in all considered cases (500 ns). During the first 10 ns of the simulation of the highest replica (with the changed interaction between Ca^2+^ ions and carboxyl groups), all calcium bridges that had formed in the classical unbiased MD simulation were destroyed. This leads to structural rearrangements in the highest replica, which should enhance statistical sampling.

#### 4.2.2. Exchange Frequencies between Replicas

**Figure 5:**
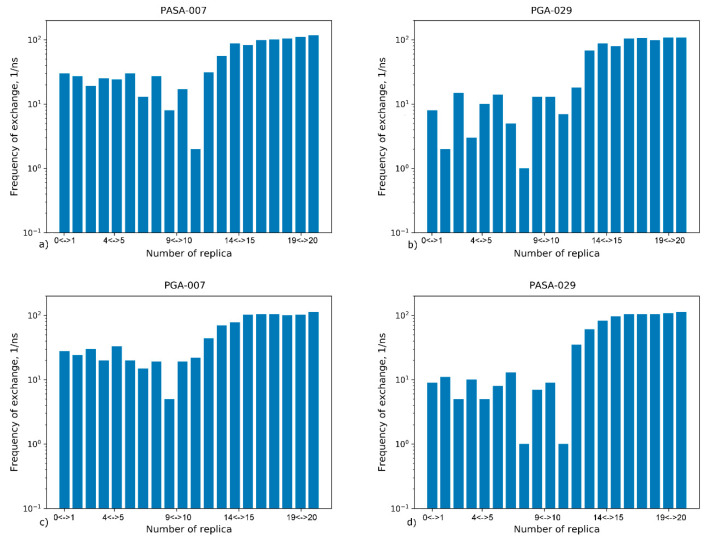
The exchange frequencies between neighbor replicas for all considered systems (**a**) PASA-0.07 mol/kg CaCl_2_ (**b**) PASA-0.29 mol/kg CaCl_2_ (**c**) PGA-0.07 mol/kg CaCl_2_ (**d**) PGA-0.29 mol/kg CaCl_2_.

The replica exchange rates between neighboring replicas during the production run were analyzed, see Figure 5.

It can be seen that exchanges occur between all neighboring replicas. Three regions can be distinguished in the distributions: (1) the lowest replicas (approximately 0 to 8), the middle replicas (approximately from 9 to 11), and the highest replicas (approximately from 12 to 20). In the region of the highest replicas, the exchange frequency between the replicas is the highest. The frequencies in the region of lowest replicas are an order of magnitude lower. The lowest frequencies are in the middle region that forms a bottleneck statistical sampling. In the case of higher concentration (0.29 mol/kg CaCl_2_), exchange occurs more slowly due to higher amounts of Ca^2+^ ions adsorbed on the chains. This results in longer living states in the region of the lowest replicas. The distribution of exchanges between replicas is directly related to the sets of conformational states in each replica. In the situation where equal steps in the Hamiltonian lead to equal changes in the structure, the distribution of exchanges between replicas will be uniform. The uneven distribution indicates a complex, non-linear dependence of the structure of the system on the Hamiltonian [98]. To increase the sampling, one can use a non-linear distribution of the α-parameter and temperature among the replicas. To enhance the exchange between replicas in the bottleneck region, the step size of these parameters can be chosen to be smaller than in other regions as has been demonstrated by Hritz et al. [98].

#### 4.2.3. Effect of the Conformational State Exchange on the Gyration Radius Distribution

**Figure 6:**
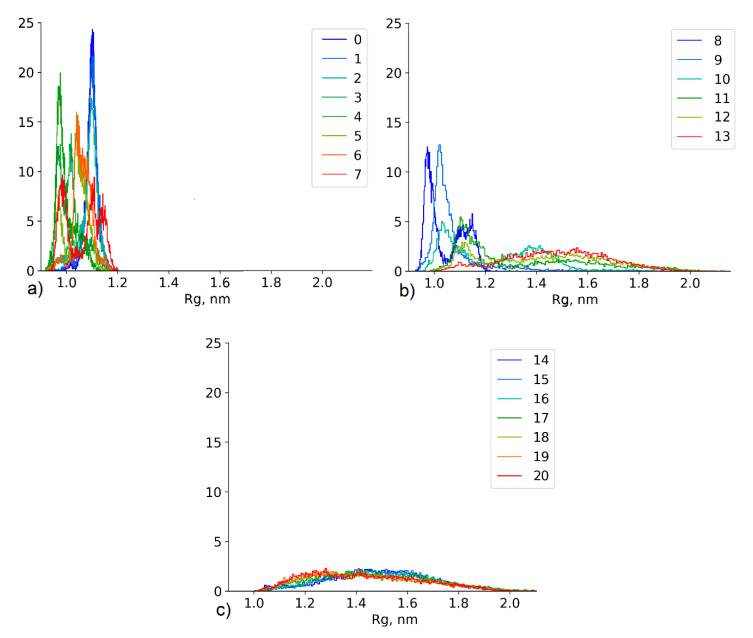
Radius of gyration distributions of PASA in 0.07 mol/kg CaCl_2_ solution for all simulated replicas (**a**) 0–7 replicas (**b**) 8–13 replicas (**c**) 14–20 replicas.

The bottleneck in the distribution of replica exchange frequency (Figure 5) is directly related to the differences in the conformations between replicas. It can be clearly seen in the case of PASA in 0.07 mol/kg CaCl_2_ solution: Figure 6 illustrates the distributions of the radius of gyration of PASA for all simulated replicas in the HRE production run.

In the highest replicas, the radius of gyration distribution is the same for all replicas. It is wide and most of the conformational states are allowed due to the fully released motions of the chain. Similar conformational states of neighboring replicas lead to a high probability of exchange (Figure 6c). The differences in the distributions of the neighboring replicas in the lowest region are higher. Calcium bridges lead to collapsed chains and low R_g_ values. The change in the dihedral potential (increased flexibility) leads to a more compact structure than in the neutral replica (Figure 6a). The differences in the conformational states between the neighboring replicas lead to lower (in comparison with the high replicas) exchange frequencies. The middle region represents the transition state of the chain (Figure 6b). The difference between the conformational states of the neighboring replicas (especially replicas 10 and 11) is the most significant and as a result, this region had the lowest exchange frequency. These replicas have the most different (between neighbor replicas) sets of conformational states.

**Figure 7:**
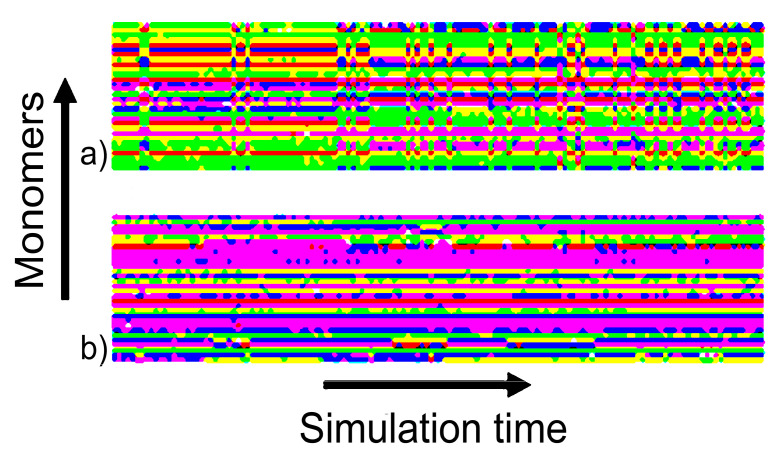
The change of the dihedral angles of the main chain in each monomer in a typical section of a trajectory obtained from (**a**) HRE simulation and (**b**) MD simulation. Each pixel illustrates the conformation of one monomer during 100 ps. The colors indicate the combination of φ and ψ dihedral angles corresponding to specific secondary structures (see Figure S2): PPII helix—pink, 2.5_10_ helix—blue, 3_10_ helix—yellow, right-handed α helix—green, left-handed α helix—red.

Figure 6 illustrates that the set of replicas generates a wide set of new conformational states, which corresponds to the radius of gyration values between ~0.9 and ~2.0 nm. However, only those states which satisfy the conditions (see Equation (3)) of exchange are able to achieve the neutral replica. Considering the active exchange of states between the replicas (Figure 5), it can be assumed that the distribution of the neutral replica corresponds to the extended statistical ensemble. The efficiency of this approach and the emergence of new states in the neutral replica (replica number 0 in Table 2) are illustrated in Figure 7. The figure shows the change of the dihedral angles of the backbone in each monomer in a typical section of a HRE trajectory in comparison with a classical unbiased MD simulation. In the case of HRE, changes occur in all monomers simultaneously. In the unbiased MD simulation, changes of monomer conformations occur independently for each monomer.

### 4.3. Results of HRE Simulation

#### 4.3.1. Chain Conformations

**Figure 8:**
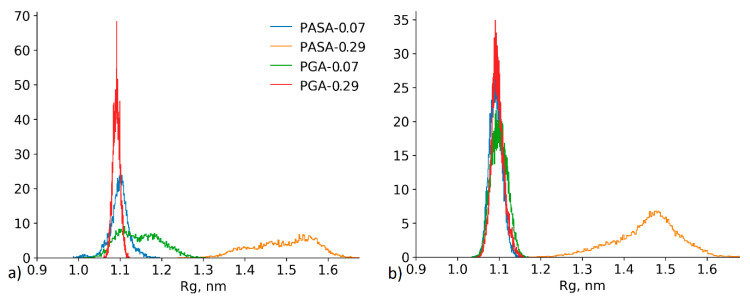
The radius of gyration distributions obtained from (**a**) HRE simulations and (**b**) classical non-biased MD simulation.

One of the main results is related to the different effects of calcium bridges on PGA and PASA chain conformations as observed in the classical MD simulations (Figure 2a). The comparison of the radius of gyration distributions from HRE and MD simulations (Figure 8) shows that the distributions are similar. The use of HRE, however, allowed distinguishing between the differences in PGA and PASA chain sizes at low CaCl_2_ concentrations.

In the case of a neutralized chain (0.07 kg/mol CaCl_2_), the chain is collapsed in both cases (PASA and PGA), due to the decrease in electrostatic repulsion between the charged carboxyl groups (as it must be [28]). The shorter PASA side chain (Figure 1) with charged carboxyl groups connected by calcium bridges allows the formation of a more compact structure than the longer PGA side chains. With the increase in concentration (to 0.29 mol/kg CaCl_2_), the PASA chain becomes more elongated due to overcharging and repulsion between the adsorbed Ca^2+^ ions by carboxyl groups (see Figure 2a). At this concentration PGA is collapsed and the distribution is very narrow (Figure 8). PGA’s longer side chains help to increase the distance between the adsorbed Ca^2+^ ions (decreasing the electrostatic repulsion between them) while maintaining a collapsed chain. In addition, the longer side chains of PGA result in lower local dielectric constant at the backbone region. Further increase in concentration should remove the differences between the chain sizes of PASA and PGA due to increased amounts of adsorbed ions and the increased screening in the solution. These results show the crucial role of the side chain length with terminal charged groups. Longer side chains make the concentration range of fully collapsed chains much wider (see Figure 2a). This is in agreement with Kundagrami and Muthukumar [28].

**Figure 9:**
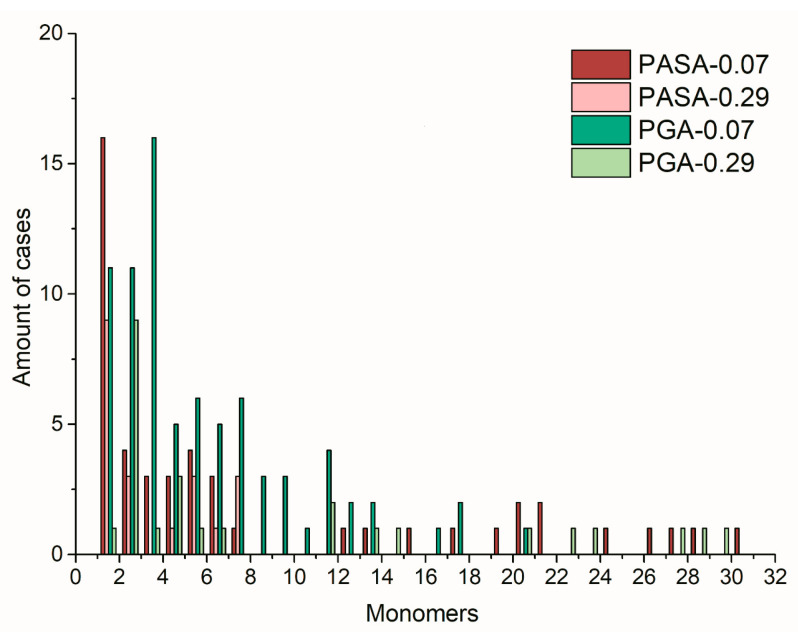
Distribution of the distances along the chain between monomers connected by calcium bridges.

The observed chain conformations are caused by the formation of calcium bridges between the carboxyl groups as can be seen from the comparison of the distributions of the distances along the chain between the monomers connected by the bridges (Figure 9). In the case of fully collapsed chains, calcium bridges connect monomers that are remote from each other. In the case of a stretched chain, the Ca^2+^ ions connect only neighbor monomers.

**Figure 10:**
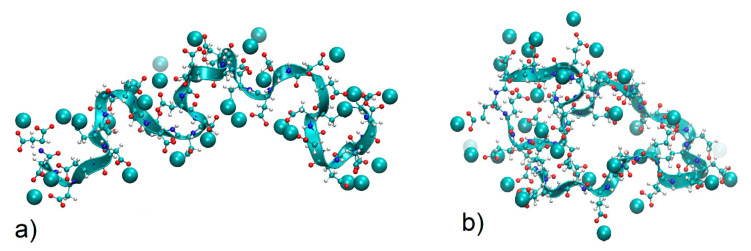
Snapshots of typical chain conformations with Ca^2+^ ions bridging the carboxyl groups. (**a**) PASA and (**b**) PGA at 0.29 mol/kg CaCl2 solution.

The distance between monomers connected by bridges determines the sizes of the loops stabilized by the calcium bridges. Bridges which connect monomers close to each other (1–3 monomers) (Figure 10b), stabilize the small loops; the bridges between remote monomers (more than 15 monomers) stabilize larger loops and have a significant effect on the radius of gyration (Figure 10a).

It can be seen from Figure 10 that the PASA chain is more linear than the PGA one. This is also confirmed by the estimations of the asphericity parameter (*b*), relative shape anisotropy (*k*^2^) and prolateness (*P*) calculated from the R_g_ tensor:

(4) $$b=\frac{R_{x}-0.5*\left(R_{y}+R_{z}\right)}{R_{x}}$$

(5) $$k^{2}=1-3\frac{R_{x}R_{y}+R_{y}R_{z}+R_{x}R_{z}}{{\left(R_{x}+R_{y}+R_{z}\right)}^{2}}$$

(6) $$P=\frac{\left(2R_{x}-R_{y}-R_{z}\right)\left(2R_{y}-R_{x}-R_{z}\right)\left(2R_{z}-R_{y}-R_{x}\right)}{2{\left(R_{x}^{2}+R_{y}^{2}+R_{z}^{2}-R_{x}R_{y}-R_{y}R_{z}-R_{x}R_{z}\right)}^{3/2}}$$

where *R_x_*, *R_y_*, *R_z_* are the principal components of the radius of gyration tensor. The values of b and *k*^2^ lie in the range from 0 to 1. The value *b* = 0 corresponds to the ideal sphere and *k*^2^ *= 0* corresponds to highly symmetric conformations and *k*^2^ = 1 to an ideal linear chain.

To get a sense of the values of prolateness, assume that *R_x_ ≥ R_y_ ≥ R*_z_ ≥ 0. When *R_y_ < (R_x_ + R_z_)*/2, prolateness is positive (i.e., the ellipsoid is prolate), and when *R_y_ < (R_x_ + R_z_)*/2, prolateness is negative (i.e., the ellipsoid is oblate). The case of *b* = 0, *P* = 0 for *R_x_* *≅ R_y_* *≅ R_z_* corresponds to a sphere-like conformation. The values are shown in Table 3.

For PASA-0.07 mol/kg, PGA-0.07 mol/kg and PGA-0.29 mol/kg, b (asphericity) is close to 0.5, the corresponding shape anisotropy is close to zero, but prolatenesses are different: in PASA-0.07 mol/kg, the chain forms an oblate ellipsoid while in PGA-0.07 mol/kg and PGA-0.29 mol/kg the chains form a prolate ellipsoid. In the system PASA-0.29 mol/kg the chain also forms prolate ellipsoid with larger asphericity and shape anisotropy.

#### 4.3.2. Local Conformations

**Figure 11:**
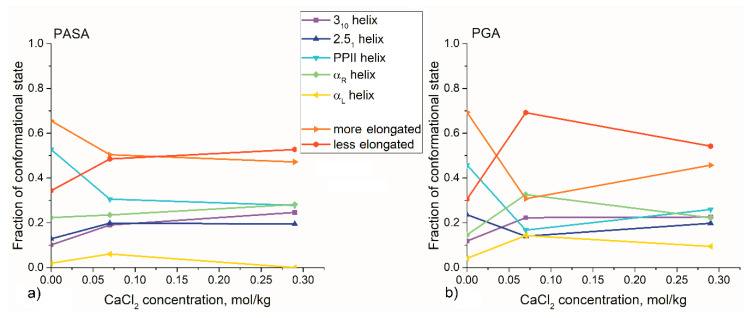
The results from the integration of the areas of the Ramachandran plots related to different secondary structures (more stretched (PPII and 2.5_1_ helices) and less stretched (α (left-handed and right-handed) and 3_10_ helices). The Ramachandran plot with the specified areas is shown in Figure S2. (**a**) PASA (**b**) PGA.

It is well-known that strong interactions such as hydrogen bonding result in alpha-helix formation in PGA and PASA chains at low pH [99,100]. To answer the question of how Ca^2+^ ions influence the secondary structures of PASA and PGA, we analyzed the changes in the dihedral angles of the chain backbone. The Ramachandran plots were obtained for each of the HRE simulations (Figure S6). The changes in the populations related to different conformational states upon increasing CaCl_2_ concentration were analyzed (Figure 11). The borders of the considered areas on the Ramachandran plot are shown in Figure S2.

It can be seen from Figure 10 that the changes of local conformations with the addition of CaCl_2_ have the same pattern for both PASA and PGA. Calcium adsorption leads to an increase in the fraction of the dihedral angles related to less elongated states (α and 3_10_ helices). The lowest fraction is observed in water and at 0.07 mol/kg the highest values. The highest contribution in less elongated states leads to a reduction in PPII conformations (Figure 11).

Another effect of CaCl_2_ on the local structure is an increased fraction of the left-handed α helices. The corresponding dihedral angles are almost absent in water due to differences between the steric hindrances of the side-chains in the left-and right-handed helices. The cause of this effect is related to the calcium bridges: The gain in energy due to the formation of an ion bridge is so large that the loss in energy due to steric hindrances in the case of left-handed helices becomes less critical. The population of the dihedral angles, which corresponds to left-handed α-helix structures, is higher for PGA in all observed cases. This can be explained by the initially greater propensity of this conformation for PGA in pure water (Figure 11).

Changes in conformations do not lead to the appearance of regular structures. Analysis of the distributions of the lengths of the regular segments of identical monomer conformations (Figure S7) shows that the chains in CaCl_2_ solution become even more irregular than in water. There are no long regular sections in the chains. All regular sections are shorter than four monomers.

#### 4.3.3. Distribution of Calcium Ions Adsorbed by PASA and PGA Chains

It has been shown previously that polyelectrolytes and amino acids can be used for the regulation of the mineral crystallization process [12,13,29,101]. At the earliest stages of mineral phase formation, an organic molecule adsorbs mineral ions forming the pattern for further crystallization process. To predict the influence of polyelectrolyte mineral morphology, the distribution of the adsorbed ions relative to each other could be used [29,74]. Kahlen et al. compared the distributions of Ca^2+^ ions adsorbed on oligomers of glutamic acid (obtained from HRE simulations) with distributions for two forms of calcium oxalate. They showed the creation of a pattern for further crystallization and demonstrated the agreement with the experimental fact that the oligomers with the different degree of polymerization (5 and 10 monomers) induce different structures of calcium oxalate [102].

**Figure 12:**
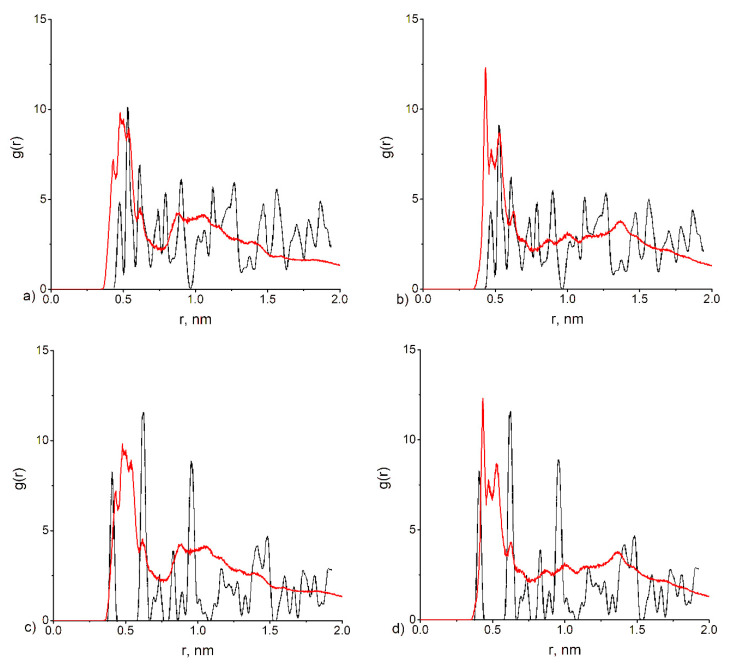
The pair distribution functions obtained from our simulations compared to the distribution obtained for calcium tri-hydrated and di-hydrated oxalates extracted from [75]. Comparison between calcium oxalate tri-hydrate (COT) and (**a**) PASA, (**b**) PGA. Comparison between calcium oxalate di-hydrate (COD) and (**c**) PASA, (**d**) PGA.

It is known that aspartic and glutamic acid influence calcium carbonate crystallization differently by suppressing crystal growth in different directions [12]. However, the effect of the poly(amino acid) type of mineral crystal cell structure has not yet been investigated to the best of our knowledge. To analyze the plausible differences in effects on the crystal structures between PASA and PGA, we investigated the pair distribution functions for Ca^2+^ ions adsorbed on the poly(amino acids) (in 0.29 mol/kg CaCl_2_ solution) (Figure 12). In addition, we compared the distributions with the distributions of the Ca^2+^ ions in calcium tri-hydrated and di-hydrated oxalates to see how our results compare to the work of Kahlen et al. [74].

No qualitative differences were observed between the PASA and PGA systems. Increase in the degree of polymerization leads to the formation of large loops as discussed above, which are stabilized by ions. These ions together with the carboxyl groups form stable clusters. Inside these clusters, the distances between the Ca^2+^ ions are much shorter and correspond to the closest distances between the Ca^2+^ ions in the crystal structure. Our results show that in the region of short distances there are peaks corresponding to calcium oxalate tri-hydrate (COT) (0.47, 0.53, 0.63, 0.88 nm) or to calcium oxalate di-hydrate (COD) (0.43, 0.63, 0.99, 1.38 nm) both for PASA and PGA. It means that long enough chains could provoke the formation of both structures. It seems plausible that the final structure of the crystal should depend on the conditions of synthesis.

## 5. Conclusions

We performed both unbiased MD and HRE simulations of anionic PGA and PASA in CaCl_2_ solutions. The formation of loops in the chain stabilized by clusters consisting of ions and carboxyl groups was observed. These clusters restrict backbone motions thus severely hindering the sampling of the statistical ensemble by classical unbiased MD. The presence of clusters leads to narrow distributions of states. This is also a challenge for HRE simulations; to overcome the problem and to simulate polyelectrolytes in multivalent salt solutions one should use a large number of replicas (here 21) to ensure successful replica exchange. The HRE simulations helped to reveal the crucial role of the poly(amino acid) side chain length. It was shown that the radius of gyration depends on CaCl_2_ salt concentration the same way as it does for semiflexible polyelectrolyte chains in a multivalent salt solution [23]. At low salt concentrations, with the addition of the salt, the chain forms an irregular collapsed globule. With a further concentration increase, the size partially recovers. Due to its longer side chain, PGA stays in the collapsed state for a wider concentration range. Therefore, PGA, at the concentration at which PASA has an extended conformation, maintains the conformation of a dense globule. This can explain the differences in mineralization of organic matrices using PASA and PGA [8]. The use of PASA leads to the formation of a polymer-induced liquid-precursor, which is able to fill the pores. On the other hand, under mineralization conditions, the use of PGA leads to the formation of mineral precursors with more compact structures and less mobile ions and these mineral precursors cannot enter into the pores of the organic matrix. In addition, our simulations are in agreement with and elaborate on the theory of Kundagrami and Muthukumar [28]: PGA chains have a lower local dielectric constant close to the backbone (due to the longer side chains) which gives rise to the above behavior. The agreement of our results with the theory [28] implies that our results are applicable in the context of longer chains that are typically used in experiments [8]. Understanding the effects of multivalent salts on polyelectrolyte conformations is also important for developing membranes based on polyelectrolyte brushes. Thus, our results suggest that changing side-chain length with the terminal charged group allows sensitivity regulation of the brush to changes in salt concentration. Grafted polyelectrolytes with longer side chains are thus able to adsorb more mineral ions in the fully collapsed form, which affects membrane penetration.

## Figures and Tables

**Table 1:** The numbers of ions used in the simulated systems.

CaCl_2_ Concentration, mol/kg	Number of Ca^2+^ Ions	Number of Cl^−^ Ions
0 (water)	0	0
0.03	6	12
0.07	14	28
0.29	58	116
0.75	145	290
1	190	380

**Table 2:** Parameters varied in the Hamiltonian replica exchange (HRE) simulations.

Replica	T, K	α	σ, nm	ε, kJ mol^−1^
0	300	0	0.273239	0.50208
1	300.75	−0.05	0.275077	0.500581
2	301.5	−0.1	0.276915	0.499083
3	302.25	−0.15	0.278753	0.497584
4	303	−0.2	0.280591	0.496085
5	303.75	−0.25	0.282429	0.494587
6	304.5	−0.3	0.284267	0.493088
7	305.25	−0.35	0.286105	0.491589
8	306	−0.4	0.287943	0.49009
9	306.75	−0.45	0.289781	0.488592
10	307.5	−0.5	0.291619	0.487093
11	308.25	−0.55	0.293457	0.485594
12	309	−0.6	0.295295	0.484096
13	309.75	−0.65	0.297134	0.482597
14	310.5	−0.7	0.298972	0.481098
15	311.25	−0.75	0.30081	0.4796
16	312	−0.8	0.302648	0.478101
17	312.75	−0.85	0.304486	0.476602
18	313.5	−0.9	0.306324	0.475103
19	314.25	−0.95	0.308162	0.473605
20	315	−1	0.31	0.472106

**Table 3:** Principle components of the radius of gyration tensor (*R_x_, R_y_, R_z_*). Asphericity parameter (*b*) (Equation (4)), relative shape anisotropy *k*^2^ (Equation (5)), and prolateness (*P*) (Equation (6)) in the systems.

	PASA-0.07 mol/kg	PASA-0.29 mol/kg	PGA-0.07 mol/kg	PGA-0.29 mol/kg
*R_x_*, nm	0.866 ± 0.037	1.391 ± 0.085	0.912 ± 0.075	0.845 ± 0.012
*R_y_*, nm	0.607 ± 0.030	0.452 ± 0.031	0.557 ± 0.034	0.563 ± 0.156
*R_z_*, nm	0.289 ± 0.032	0.292 ± 0.046	0.409 ± 0.043	0.399 ± 0.016
*(R_y_ + R_z_)*/2, nm	0.578 ± 0.022	0.842 ± 0.032	0.660 ± 0.34	0.622 ± 0.008
*B*	0.481 ± 0.039	0.731 ± 0.030	0.466 ± 0.062	0.430 ± 0.014
*k* ^2^	0.082 ± 0.014	0.233 ± 0.037	0.059 ± 0.020	0.047 ± 0.005
*P*	−0.169 ± 0.266	0.911 ± 0.056	0.603 ± 0.293	0.435 ± 0.154

## Supplementary Materials

The following are available online at https://www.mdpi.com/2073-4360/12/6/1279/s1. Figure S1: Number of adsorbed calcium ions in classic unbiased MD simulations. Figure S2: Ramachandran plot showing of the areas of angles related to peptide secondary structures and transitions between them. Description of the fitting of potential mean force of backbone dihedral angles. Figure S3: The potential of mean force of backbone dihedral angles and the results of the fitting. Figure S4: Dihedral potentials used in the HRE simulations from 0 to 20 replicas, Table S1: Parameters obtained from fitting. Description of the procedure for calculation of the lifetimes of Ca^2+^ bridges. Figure S5: Radial distribution functions between the oxygens of the different carboxyl groups for the highest replica. Figure S6: The Ramachandran plots for PASA and PGA in calcium chloride solution from the HRE simulations. Figure S7: The distributions of the lengths of the regular segments of monomer conformations.
