# Supplementary material for: Influence of Calcium Binding on Conformations and Motions of Anionic Polyamino Acids. Effect of Side Chain Length
Source: Polymers (Basel). 2020 Jun 3;12(6):1279. doi: 10.3390/polym12061279 (PMC7362111; doi:10.3390/polym12061279)
Supplement: Supplementary file 1 [file polymers-12-01279-s001.pdf]

Supplementary Information

Influence of Calcium Binding on Conformations and Motions of Anionic Polyamino Acids. Effect of Side Chain Length

Dmitry Tolmachev [1,*], Natalia Lukasheva [1], George Mamistvalov [2] and Mikko Karttunen [1,3,4,5,*]

1 Institute of Macromolecular Compounds, Russian Academy of Sciences, Bolshoy pr. 31, 199004 St. Petersburg, Russia; luk@imc.macro.ru
2 Faculty of Physics, St. Petersburg State University, Petrodvorets, 198504 St. Petersburg, Russia; mamistvalov.georgii@gmail.com
3 Department of Chemistry, the University of Western Ontario, 1151 Richmond Street, London, ON N6A 5B7, Canada
4 Department of Applied Mathematics, the University of Western Ontario, 1151 Richmond Street, London, ON N6A 5B7, Canada
5 The Centre of Advanced Materials and Biomaterials Research, the University of Western Ontario, 1151 Richmond Street, London, ON N6A 5B7, Canada
* Correspondence: dm.tolmahev@yandex.ru (D.T.); mkarttu@uwo.ca (M.K.)

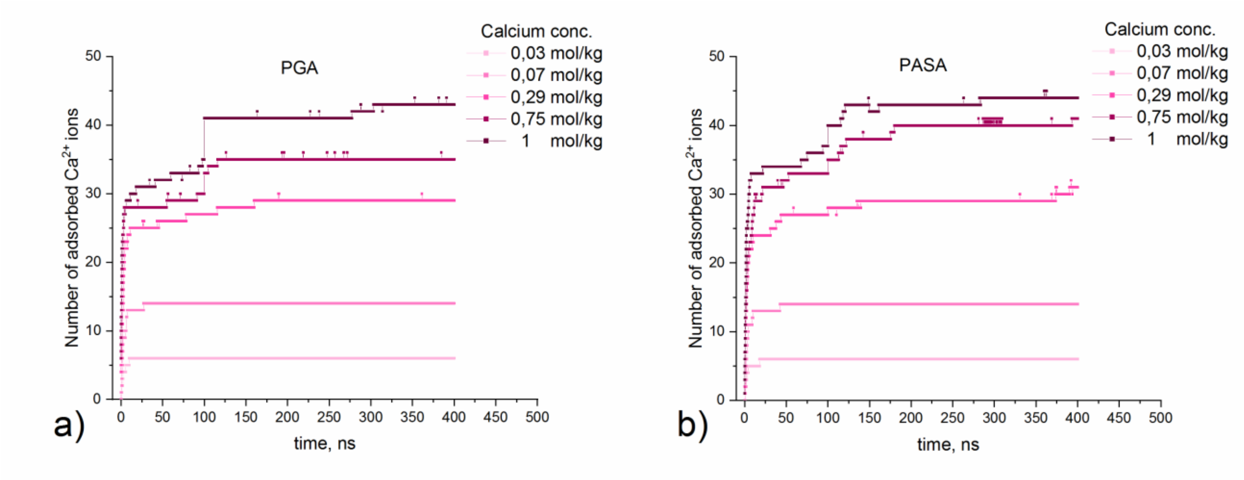

Figure S1. Number of adsorbed calcium ions on a) PGA and b) PASA molecules in classic unbiased MD simulations.

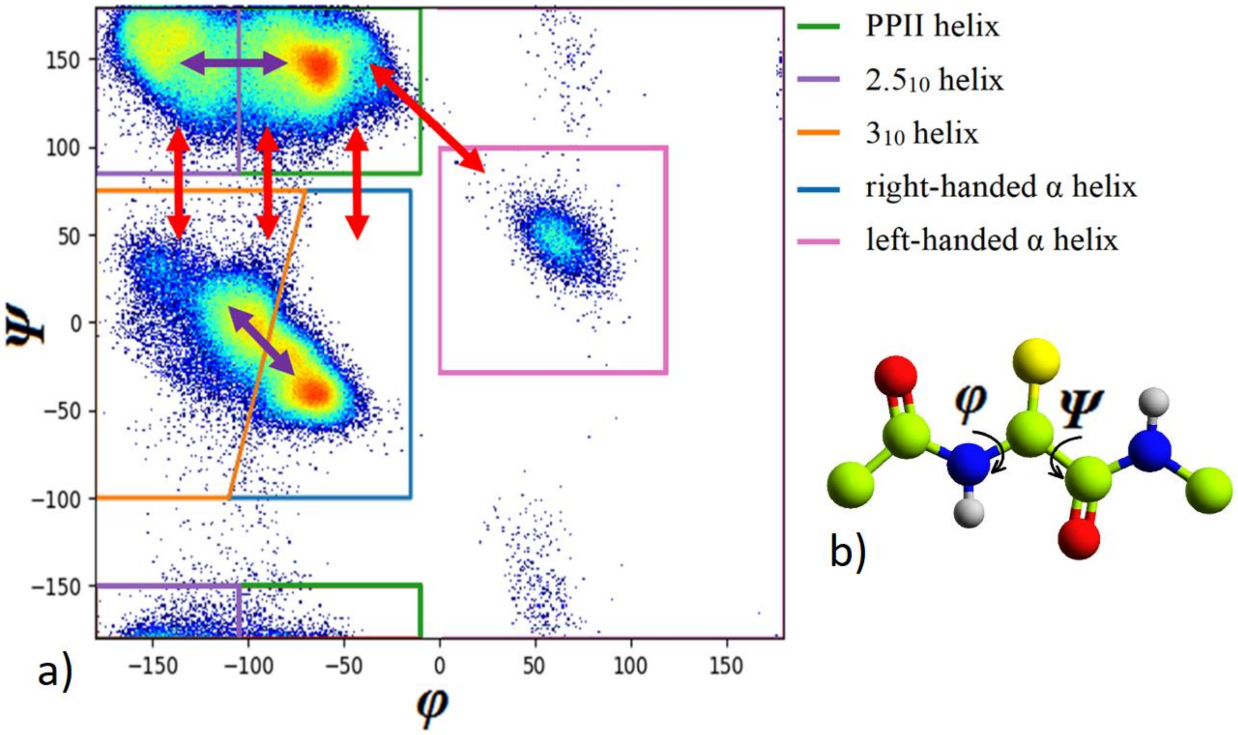

Figure S2. a) Ramachandran plot showing of the areas of angles related to peptide secondary structures and transitions between them. Transitions through low-energy barriers: red arrows, transitions through high-energy barriers: violet arrows). b) Amino acid residue with dihedral angles ψ and φ (green: carbon, blue: nitrogen, white: hydrogen, yellow: side chain) indicated.

Potential mean force of dihedral angles ψ and φ.

The potential of mean force (PMF) was obtained based on the distributions of the dihedral angles obtained in simulations of the PASA and PGA in water by equation:

$$\mathrm{PMF} = -\mathrm{k_b T} \cdot \ln(\mathrm{D}), \qquad \text{(S1)}$$

where D is the distribution of the dihedral angle, k_b is the Boltzmann coefficient, and T is temperature.

To enable implementation of the fitted results in the force field, fitting was performed with the same form for dihedral potential as in CHARMM27:

$$y = \sum_{n=1}^{15} k_n * (1 + \cos(n * x - a_n)) \qquad \text{(S2)},$$

where k_n, a_n are varied parameters.

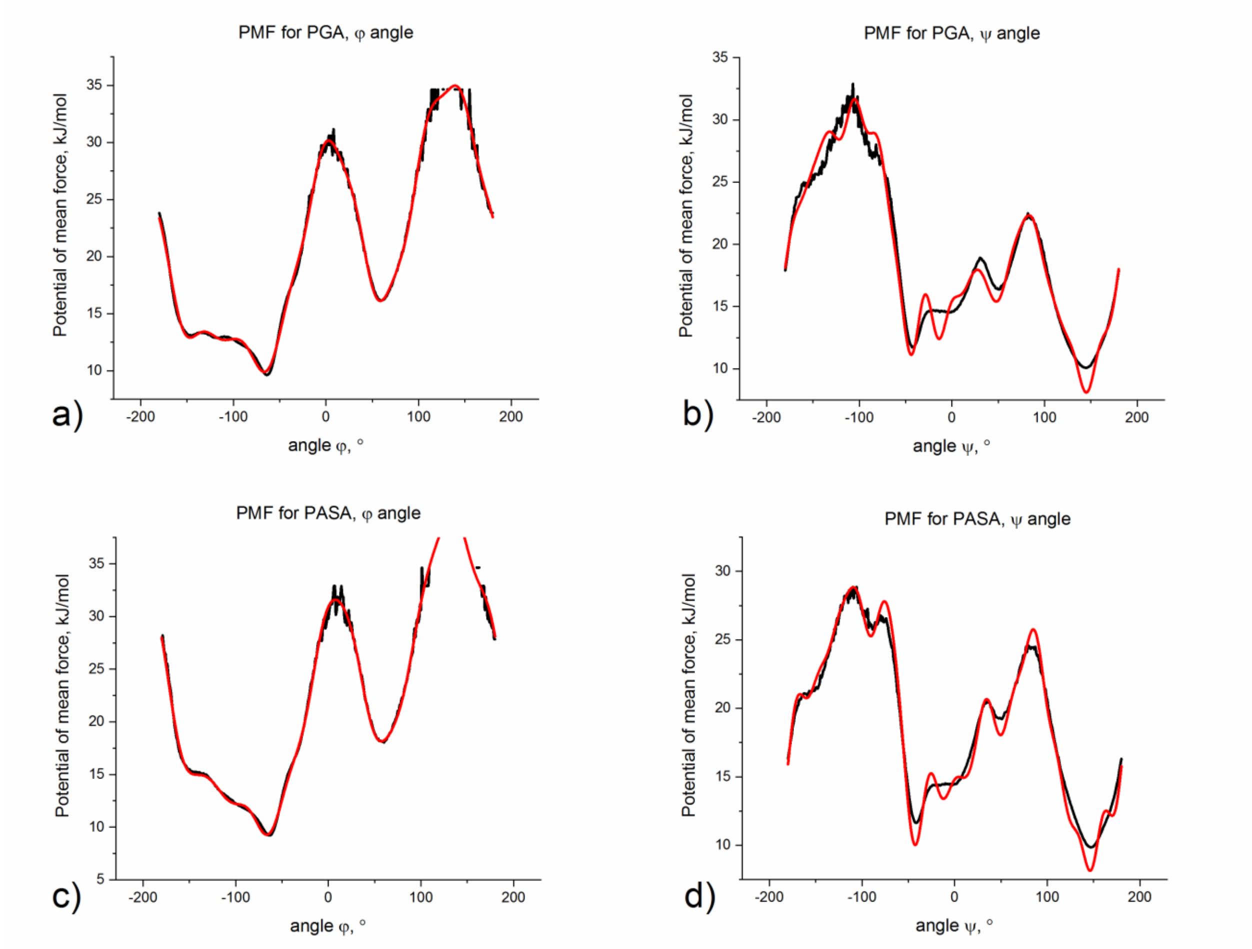

Figure S3. The potential of mean force of dihedral angle of the acid backbone (black). The result of the fitting by Eq. S1 (red). PGA in water solution: PMF of angles a) φ and b) ψ. PASA in water solution: PMF of angles c) φ and d) ψ with fitting curves.

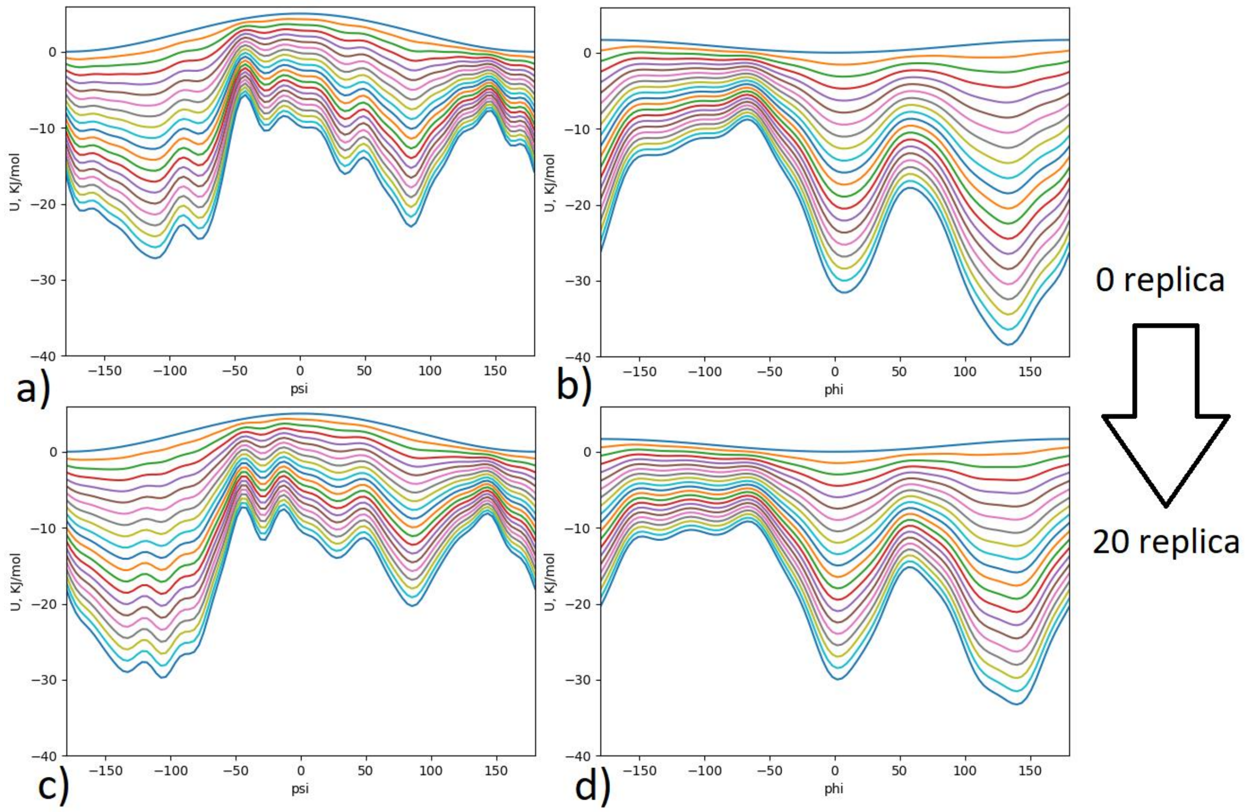

Figure S4. All dihedral potentials used in the HRE simulations from 0 to 20 replicas. For PASA: a) angle φ b) angle ψ. For PGA: c) angle φ d) angle ψ.

Table 1. Parameters obtained from fitting.

	Glutamic acid φ angle (a)		Glutamic acid ψ angle (b)		Asparatic acid φ angle (c)		Asparatic acid ψ angle (d)	
n	k_n, KJ/mol	n ,°	k_n, KJ/mol	a_n ,°	k_n, KJ/mol	a_n ,°, KJ/mol	k_n, KJ/mol	a_n ,°, KJ/mol
1	7.78832	95.9733	5.34967	-109.959	9.86809	104.2136	3.14451	-97.4951
2	6.02362	-46.2228	6.74077	127.5805	6.42604	-42.7432	6.95597	134.8657
3	4.89077	25.16316	0.86806	-51.6516	5.08541	27.57474	0.60418	-122.477
4	0.86884	11.44942	2.2176	18.50367	1.13579	22.68168	2.05245	17.86482
5	0.36728	139.4207	0.28941	-39.6177	-0.54231	-70.6703	0.3713	36.98844
6	0.01133	151.2356	0.6006	172.1778	0.29152	21.88928	0.53797	-115.222
7	0.12675	113.8691	0.90847	179.9087	-0.43642	-39.2075	1.5937	-127.954
8	0.32607	29.58869	-0.38396	3.933355	0.50066	3.516815	0.78692	-176.422
9	0.45864	-85.5884	0.36384	87.8625	0.21333	-126.867	0.43258	66.8997
10	0.28463	-1.26681	0.46984	60.74613	0.28875	-64.2142	0.50599	60.87104
11	-	-	0.48624	-26.104	-	-	0.51523	-23.2753
12	-	-	0.29617	59.26962	-	-	0.35803	64.36207
13	-	-	0.34565	-7.15395	-	-	0.35972	-6.80387
14	-	-	0.26459	-20.5342	-	-	0.29835	82.68125
15	-	-	0.29515	-117.442	-	-	0.33762	-107.407

Procedure for calculation of the lifetimes of Ca^{2+} bridges. As a criterion of calcium bridges, the distance between the oxygens of the different carboxyl groups was chosen. To calculate the lifetime of a calcium bridge, changes in the distances between the oxygen atoms of all carboxyl groups were analyzed with the time interval of 100 ps. If the distance between the carboxyl oxygens becomes

smaller than the distance corresponding to the first minimum of the radial distribution function (RDF), we defined this contact as an ion bridge. The lifetime of calcium bridge was considered as the length of the time interval on the trajectory in which the distance between the oxygens of the different carboxyl groups is lower than first minimum of the RDF between the carboxyl oxygen atoms. Figure S5 shows an example of the RDFs for the highest replica.

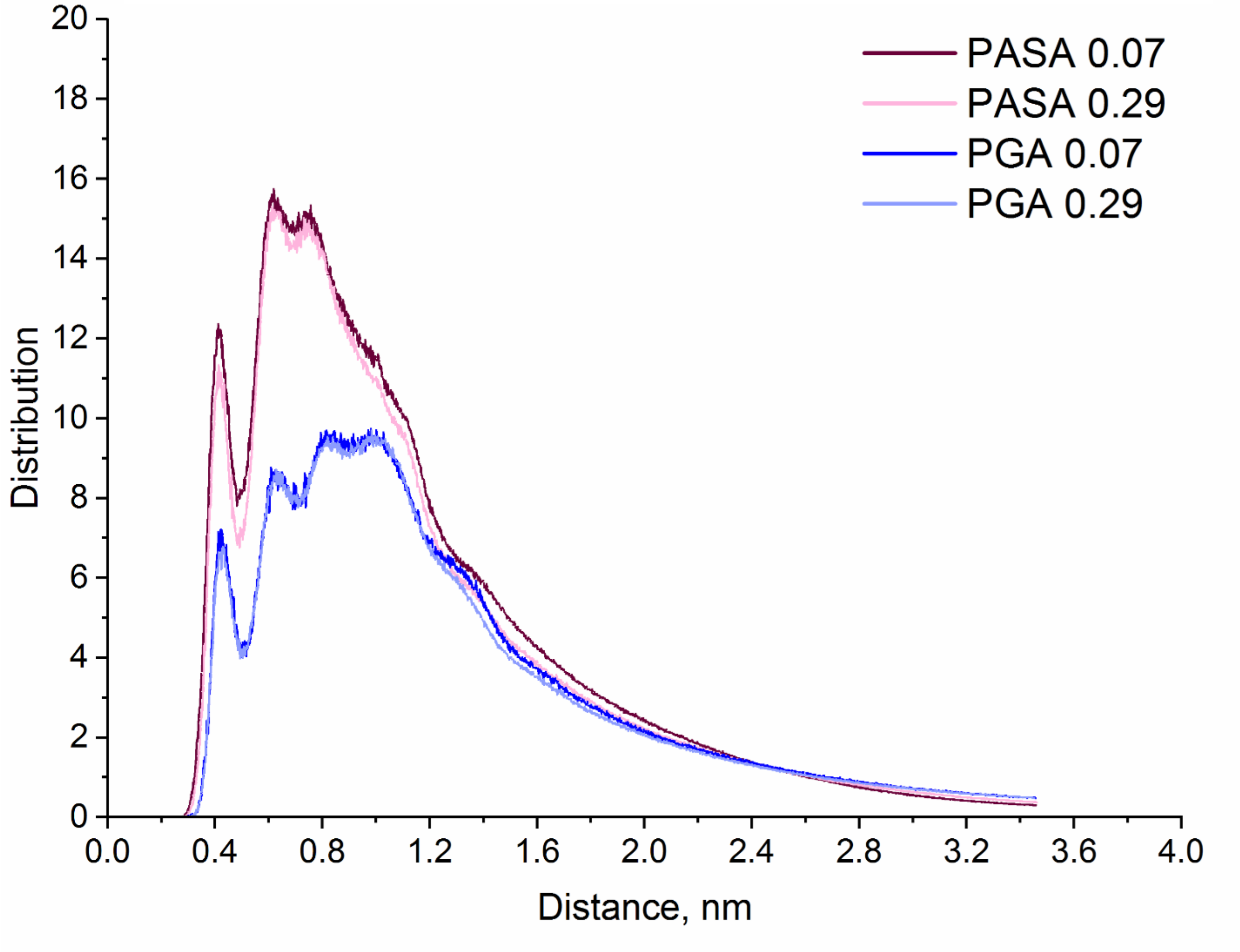

Figure S5. RDFs between the oxygens of the different carboxyl groups for the highest replica.

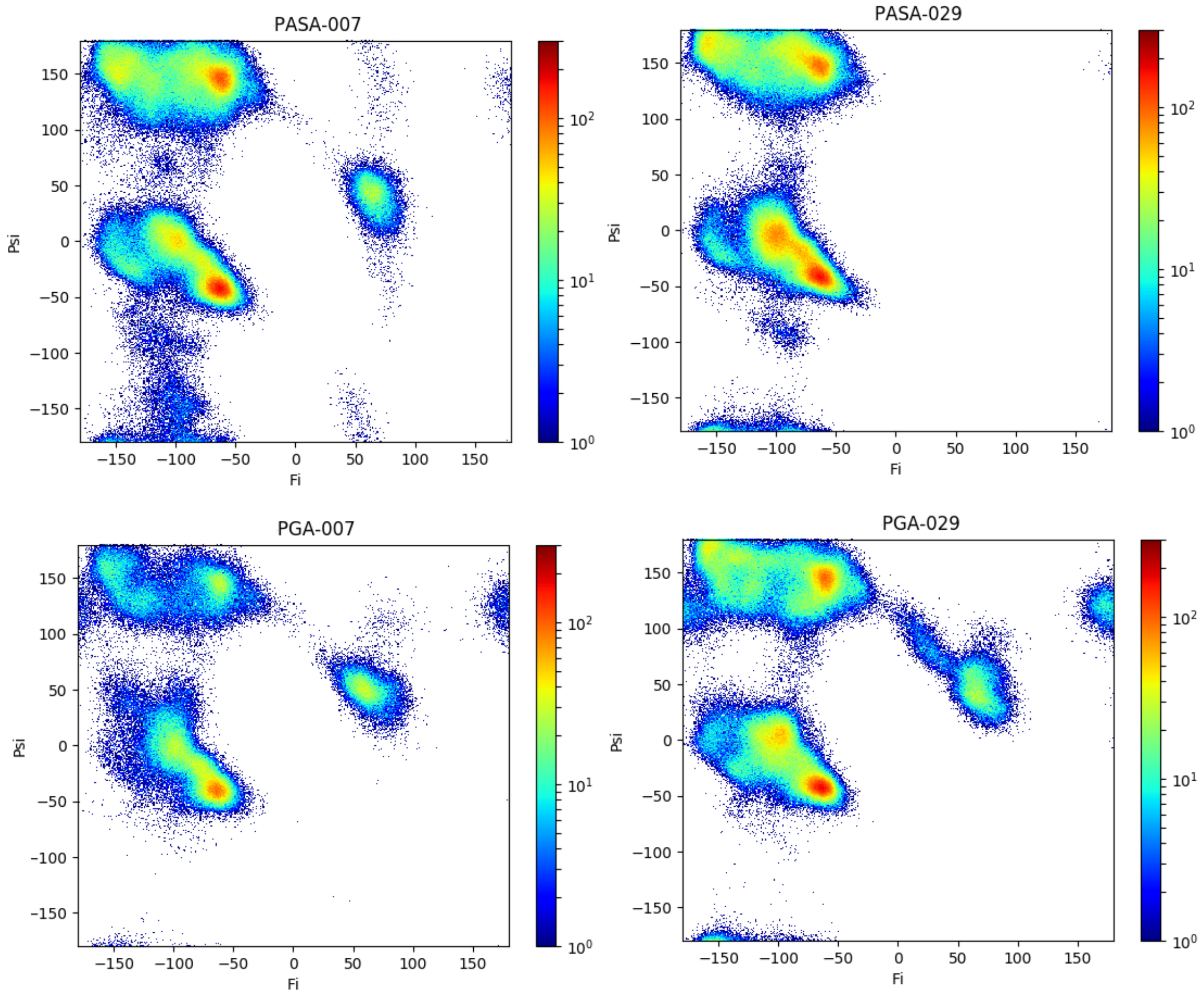

Figure S6. The Ramachandran plots for PASA and PGA in calcium chloride solution from the HRE simulations.

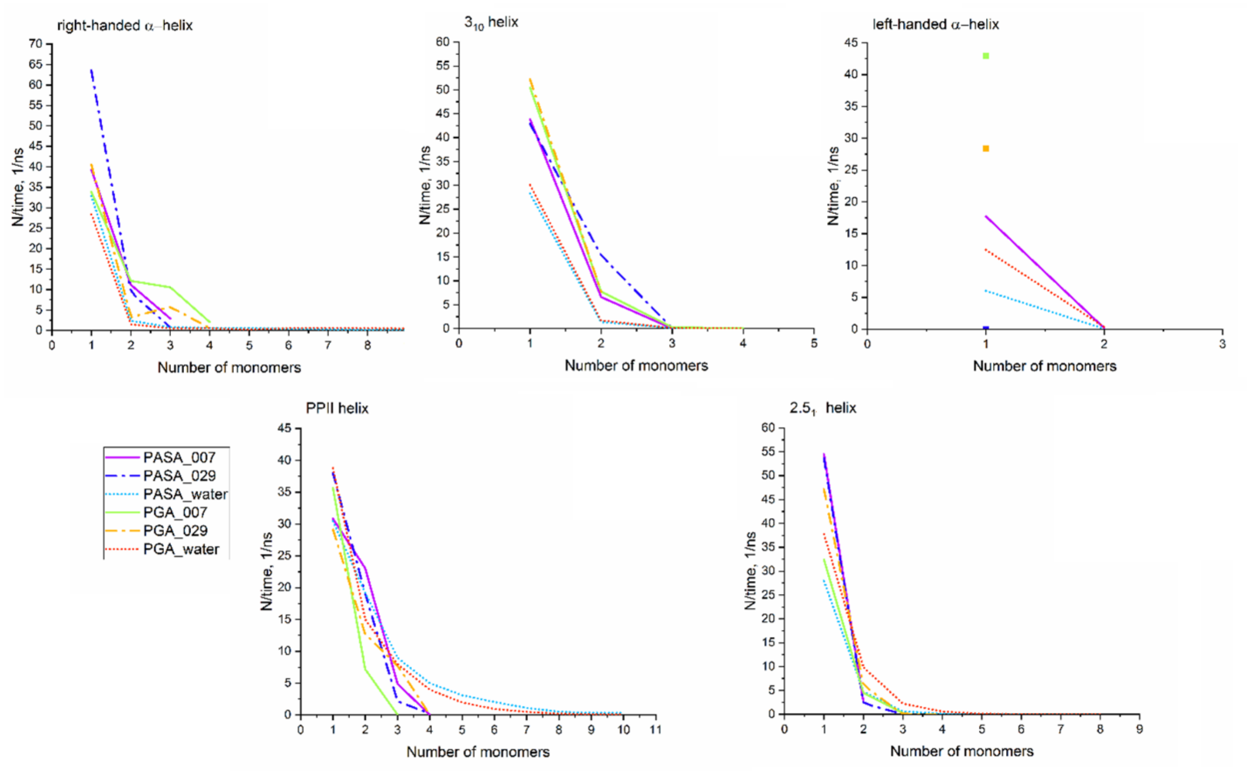

Figure S7. The distributions of the lengths of the regular segments of monomer conformations.
